# *PACLOBUTRAZOL-RESISTANCE* Gene Family Regulates Floral Organ Growth with Unequal Genetic Redundancy in *Arabidopsis thaliana*

**DOI:** 10.3390/ijms20040869

**Published:** 2019-02-17

**Authors:** Kihye Shin, Inhye Lee, Eunsun Kim, Soon Ki Park, Moon-Soo Soh, Sumin Lee

**Affiliations:** 1 Division of Integrative Bioscience and Biotechnology, College of Life Science, Sejong University, Seoul 05006, Korea; kihyeshin@hotmail.com (K.S.); ihlee44@gmail.com (I.L.); euntton@gmail.com (E.K.); 2 Present address: Basic Forestry and Proteomics Center (BFPC), Fujian Provincial Key laboratory of Haixia applied plant systems biology, Haixia institute of Science and Technology (HIST), Fujian Agriculture and Forestry University, Fuzhou 350002, China; 3 School of Applied Biosciences, Kyungpook National University, Daegu 41566, Korea; psk@knu.ac.kr

**Keywords:** *Arabidopsis thaliana*, PRE, fertility, unequal redundancy, autogamous reproduction

## Abstract

A *PACLOBUTRAZOL-RESISTANCE* (*PRE*) gene family, consisting of six genes in *Arabidopsis thaliana*, encodes a group of helix-loop-helix proteins that act in the growth-promoting transcriptional network. To delineate the specific role of each of the *PRE* genes in organ growth, we took a reverse genetic approach by constructing high order *pre* loss-of-function mutants of *Arabidopsis thaliana*. In addition to dwarf vegetative growth, some double or high order *pre* mutants exhibited defective floral development, resulting in reduced fertility. While *pre2pre5* is normally fertile, both *pre2pre6* and *pre5pre6* showed reduced fertility. Further, the reduced fertility was exacerbated in the *pre2pre5pre6* mutant, indicative of the redundant and critical roles of these PREs. Self-pollination assay and scanning electron microscopy analysis showed that the sterility of *pre2pre5pre6* was mainly ascribed to the reduced cell elongation of anther filament, limiting access of pollens to stigma. We found that the expression of a subset of flower-development related genes including *ARGOS*, *IAA19*, *ACS8,* and *MYB24* was downregulated in the *pre2pre5pre6* flowers. Given these results, we propose that PREs, with unequal functional redundancy, take part in the coordinated growth of floral organs, contributing to successful autogamous reproduction in *Arabidopsis thaliana.*

## 1. Introduction

Coordinated organ growth is essential for the determination of organ architecture and its biological functions. The process of pollination in autogamous plants is a good example to study how proper organ growth is critical for the function of organs. For successful pollination, the coordinated growth of anther filament and style, as well as the release of functional pollens, is essential. Thus filament growth and anthesis must be tightly controlled and keep pace with the pistil development for successful fertilization. Indeed, dysregulated growth of these floral organs such as shortened anther filament or longer style, termed as heterostyly, results in allogamous reproduction, requiring cross-pollination [1,2,3].

Organ growth is driven by cell division and elongation, which are controlled by integrated signals from environments and the endogenous development program mediated by plant hormones. For example, the stamen development is controlled by multiple phytohormones including GA, BLs, auxins, and JA [4,5,6,7]. Deficiency of those hormones causes male sterility [4,6,8,9,10,11,12,13,14]. The hormonal signals are either converged or independently transduced to a transcriptional regulatory network, involving transcription factors such as MYB21/MYB24 and ARF6/ARF8. In support, the mutants of *myb21myb24* or *arf6arf8* display severe sterility, primarily due to reduced floral organ growth [11,15,16]. The expression of *MYB21*/*MYB24* is regulated by multiple plant growth hormones including GA, JA, and auxin in part with a hierarchical relationship. GA and ARF6/ARF8 regulate JA-biosynthetic genes to regulate the production of JA, which in turn activates the expression of *MYB21/MYB24* [15,16]. 

A small gene family, designated as *PRE*s (*PACLOBUTRAZOL-RESISTANCE*), encode atypical helix-loop-helix (HLH) proteins in higher plants. In *Arabidopsis thaliana*, *PRE* family comprise six genes, including *PRE1/BNQ1(BANQUO1)*, *PRE2/BNQ2*, *PRE3/BNQ4/ATBS1(ACTIVATION-TAGGED bri1 SUPPRESSOR1)*/*TMO7 (TARGET OF MP7*), *PRE4/BNQ3*, *PRE5*, *PRE6*/*KDR (KIDARI)* [17,18,19,20,21,22]. Since *PRE1* has been initially characterized as a positive regulator of GA signaling [18], subsequent studies have elucidated that PREs are also involved in growth regulation by a wide range of signals, including BR, auxin, high temperature and light [20,21,22,23,24,25]. Elevated expression of any single *PRE* gene confers elongated hypocotyls/petioles, while downregulation of multiple *PRE* genes by gene silencing caused dwarf phenotypes, suggestive of functionally redundant roles of PREs to promote cell elongation. How PRE functions in cell elongation has been revealed by interactome studies [17,21,22,23,26]. Yeast two-hybrid screening of PRE interacting proteins has identified distinct classes of atypical HLH proteins such as IBH1/AIFs, PAR1/PAR2, and HFR1. These atypical HLH proteins were shown to be capable of inhibiting cell elongation, presumably through heterodimerization with bHLH proteins with growth-promoting activity, including HBI1/ACEs, BEEs or PIFs, respectively. Based on these results, it has been postulated that PRE functions in the PRE-IBH1-HBI1/ACEs or PRE-PAR1/PAR2/HFR1-PIF tripartite HLH/bHLH modules for transcriptional reprogramming, resulting in cell elongation.

Most of the functional studies of *PRE* genes have been based on the transgenic plants that ectopically express each of the *PRE* genes or transgenic plants with gene silencing of multiple *PRE*s, hampering the assessment of the function of individual PREs [17,18,19,20,25]. Thus, the in vivo function of individual *PRE* gene member remains to be determined. Despite functional similarity or redundancy among *PRE* genes, the affinity toward its interacting proteins appeared to be differential [21,27]. Besides, expression analysis of the differential inducibility of *PRE* genes by GA, auxin, or BR as well as differential tissue-preferential expression pattern implicated distinct roles of *PRE* genes [18,22,25,28]. Indeed, under a specific environmental condition or developmental context, a single *pre* mutant could exhibit developmental alterations [19,20,22]. However, redundant roles among *PRE*s still remain to be dissected through systematic high order loss-of-function mutant analysis.

In the present study, we generated multiple combinations of loss-of-function *pre* mutants and investigated the defect in organ growth in *Arabidopsis thaliana*. While single and a subset of double *pre* mutants did not show discernible growth defects, some combination of high order *pre* mutants showed shortened hypocotyls/petioles, recapitulating the known role of PREs during vegetative organ growth. Further, we also found that certain combination of *pre* mutations, including *pre2pre6*, *pre5pre6*, and *pre2pre5pre6* caused shortened anther filaments, resulting in failure in pollination. Microscopic analysis and gene expression analysis indicated that the reduced fertility of high-order *pre* mutants is due to reduced cell elongation of anther filament, which is accompanied by altered transcriptional expression of several flower-development related genes, including *ARGOS*, *ACS8*, *IAA9*, and *MYB24*. By comparing phenotypes of different combinations of high order *pre* mutants, we suggest that *PRE* gene family members take part, in an unequally redundant manner, in the regulation of cell elongation/expansion for diverse aspects of growth and development.

## 2. Results

### 2.1. Unequally Redundant Role of PREs during Vegetative Organ Growth

A small gene family, designated as *PRE* (*PACLOBUTRAZOL RESISTANCE*), has been characterized based on gain-of-function studies [17,18,19,20,21,22]. Functional redundancy among *PRE* genes was inferred by the findings that the transgenic plants that overexpressed any single *PRE* member displayed similar developmental alterations. In support of the functional similarity among PRE members, we found that all of the PRE members in *Arabidopsis thaliana* could interact with PAR1 or PAR2, a class of PRE-interacting HLH proteins [29], in yeast two-hybrid assay (Figure S1).

Based on differential spatiotemporal and hormone-responsive expression patterns of *PRE*s (Figure S2) [18,20,22,23], we hypothesized that each PRE protein plays a specified, if redundant, role during various stages of growth and development, which might constitute an evolutionary selection pressure to preserve multiple, functionally redundant [17,18,21], *PRE* genes. Indeed, a few SNP/deletion/insertion variations with a high impact were present in the *PRE* genes among 1,135 natural accessions of *Arabidopsis thaliana* (Table S1) [30]. To elucidate the function of *PRE* gene members in *Arabidopsis thaliana*, we obtained T-DNA insertional mutants of each *PRE* gene from The Arabidopsis Information Resource database (TAIR, www.arabidopsis.org). By RT-PCR analysis, we found that mutants of *pre2*, *pre5*, or *pre6* were a null allele, while the mutant of *PRE4* was a hypomorphic allele, though it harbors T-DNA in its intron (Figure S3). The *pre1* mutant was not studied further since we found that a *PRE1* T-DNA mutant (GK646C02) contains a T-DNA insertion in the 3′-UTR region, which did not alter the transcript level. For the *PRE3* gene, there is no available T-DNA insertional mutant for the present. 

**Figure 1:**
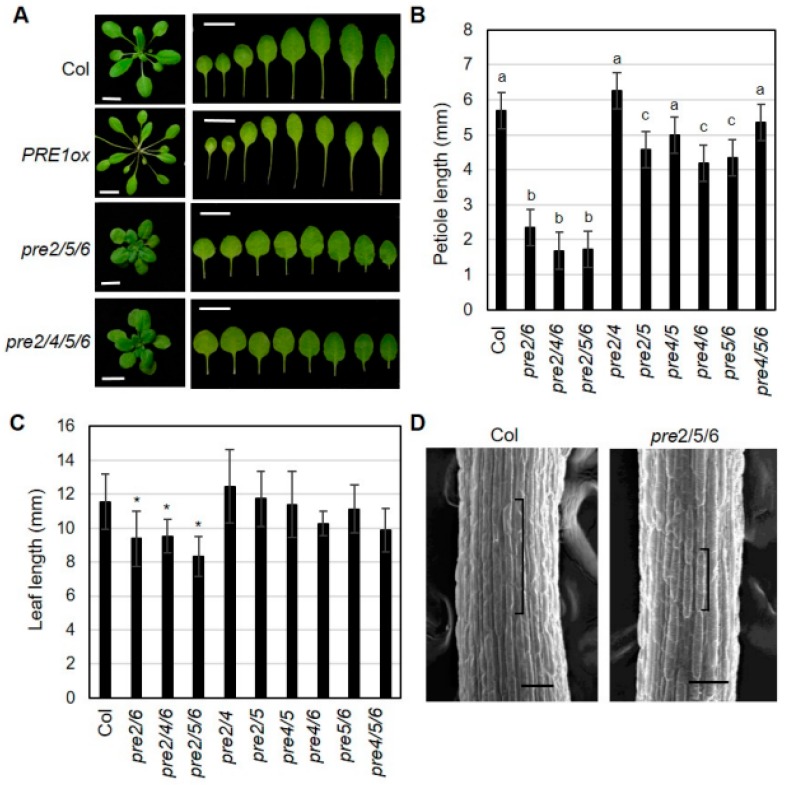
High order loss-of-function of *PRE*s alters leaf growth. (**A**) Representative leaf morphology of *pre* mutants. The pictures were taken from 3 week-old plants. The leaves were arranged according to the age from left to right. Scale bar, 1 cm. (**B**) Petiole length of *pre* mutants. The petiole length was measured with the 7th leaves of 3 week-old plants grown under long-day (16h light/8h dark). Values are mean ± SD (*n* = 12–15). Significant difference among wild type (Col) and mutants are indicated with letters (ANOVA, Tukey HSD test, *p* < 0.05). (**C**) Leaf length was analyzed from the same leaves as described in B. Values are mean ± SD (*n* = 12–15). A significant difference between wild type (Col) and mutants is indicated with asterisks (*t*-test, * *p* < 0.01). (**D**) Cell length of petiole epidermis. Petioles of the third leaf of wildtype (Col) or *pre2pre5pre6* were subject to scanning electron microscopy. Brackets indicate the lengths of single epidermal cells from the middle of petioles. Scale bar, 100 μm.

To dissect the functional redundancy among *PRE* genes, we systematically generated a series of high order *pre* mutants including double mutants of *pre2pre5*, *pre2pre6*, *pre2pre4*, *pre4pre5*, *pre4pre6*, or *pre5pre6*, triple mutants of *pre2pre4pre6*, *pre2pre5pre6*, or *pre4pre5pre6* and quadruple mutant of *pre2pre4pre5pre6*. In the course of generating the high order double mutant series, we observed several developmental abnormalities in certain combinations of *pre* mutations, revealing unequal redundancy among *PRE* genes. In agreement with the results of RNAi-based silencing of multiple *PRE*s [23,25], high order *pre* mutants, e.g., *pre2pre5pre6* exhibited a dwarf growth phenotype and reduced hypocotyl elongation under light condition (Figure 1 and Figure S4). Direct comparison of the leaf development of single, double, triple and quadruple *pre* mutants showed that *PRE2* and *PRE6* play dominant roles during vegetative growth. As shown in Figure 1, petiole growth was significantly reduced in the *pre2pre6* mutant. On the contrary, the shortened petiole was not observed or marginally changed in other double mutant series, including *pre2pre4, pre2pre5*, *pre4pre5*, *pre4pre6* and *pre5pre6*, and a triple mutant, *pre4pre5pre6*. Notably, the dwarf growth of *pre2pre6* was not exacerbated in the triple mutants of *pre2pre4pre6*, *pre2pre5pre6*. The dwarf growth of *pre2pre5pre6* was also comparable to that of the quadruple mutant, *pre2pre4pre5pre6* (Figure 1A). The leaf length, but not leaf width, was also significantly reduced in the *pre2pre6* double mutant and triple mutants, including *pre2pre6*, *pre2pre4pre6* and *pre2pre5pre6*, resulting in orbicular leaf shape (Figure 1A,C). These results implicate the dominant roles of PRE2 and PRE6 during leaf development. The scanning electron microscopy analysis showed that the cell length was clearly reduced in the petioles of *pre2pre5pre6* compared to wild type, suggesting that the shortened petiole phenotype is mainly due to reduced cell elongation (Figure 1D). 

As *PRE* genes have been implicated in the hypocotyl elongation, we also examined hypocotyl growth of high order *pre* mutant series. As shown in Figure S4, *pre2pre4pre6*, *pre2pre5pre6* and *pre2pre4pre5pre6*, but not *pre4pre5pre6*, showed shortened hypocotyl growth under light condition. Among the double mutant series, only *pre2pre6* displayed significantly shorter hypocotyl phenotype, compared to wild type, which is in line with the prominent role of *PRE2* and *PRE6* in the petiole elongation. However, we could not see any noticeable defects in the dark-grown seedlings, even in the *pre2pre4pre5pre6* quadruple mutant (Figure S4). Unlike the aerial part of vegetative organs, high order *pre* mutants exhibited normal root growth phenotype as well as normal flowering phenotypes, while *PRE1* overexpressing mutants displayed longer root length and early-flowering phenotypes, compared to wild type plant (Figure S5). These results implicate the function of other *PRE* members, *PRE1* and *PRE3,* may still support normal root growth and flowering phenotypes.

### 2.2. Unequally Redundant Role of PREs during Flower Maturation

**Figure 2:**
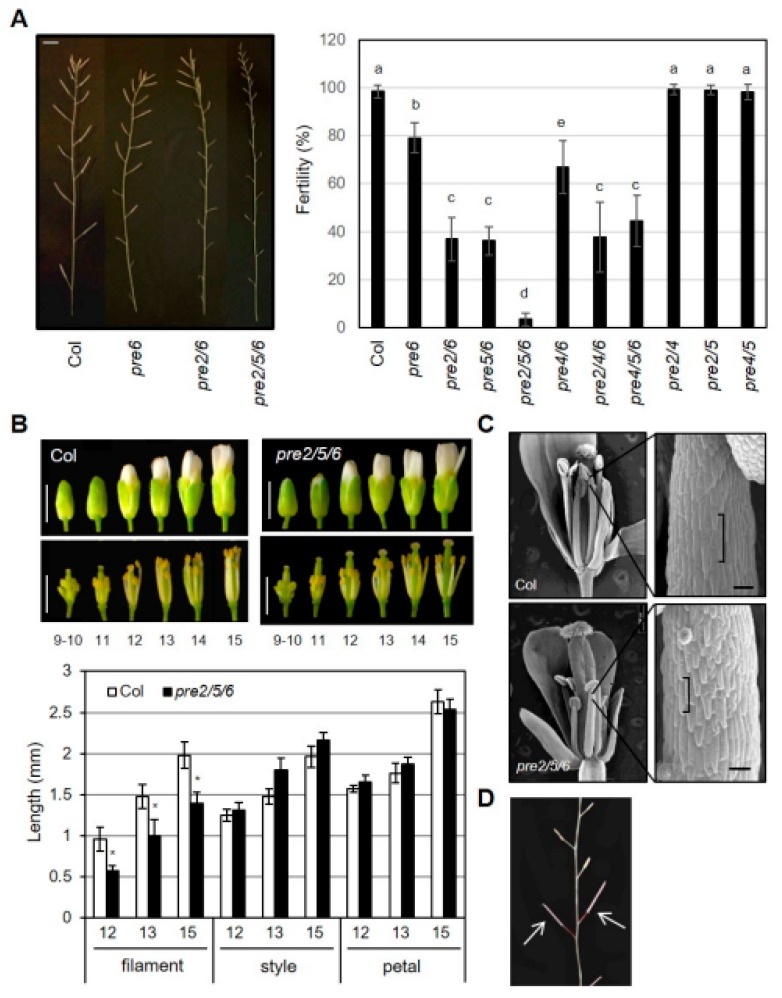
High order loss-of-function *PRE*s causes sterility. (**A**) Fertility of *pre* mutants. Primary inflorescence (left) and fertility of *pre* mutants (right). The fertility was measured as the percentage of mature siliques per whole siliques of the primary inflorescence. Values are mean ± SD (*n* = 13–15). Significant difference among wildtype (Col) and mutants are indicated with letters (ANOVA, Tukey HSD test, *p* < 0.01). Scale bar, 5 mm. (**B**) Floral organ growth of wildtype (Col) and *pre2pre5pre6* during flower development. Upper, Representative flower morphology at indicated flower developmental stages. Scale bar, 1 mm. Lower, The length of the filament, style, petal was measured at indicated flower developmental stages. Values are mean ± SD (*n* = 15). Asterisk indicates significant difference from wild type (*t*-test, * *p* < 0.01). (**C**) Reduced cell size in *pre2pre5pre6* anther filament. Scanning electron microscopy was performed on anther filament from flowers at stage13. Brackets indicate the lengths of single epidermal cells of anther filaments. Scale bar, 100 μm. (**D**) Restored fertility of *pre2pre5pre6* by self-pollination. The *pre2pre5pre6* flowers which were manually pollinated with their pollens produced fully mature siliques, as indicated by arrows.

In addition to vegetative organ growth, we observed defects of reproductive organ growth in specific combinations of *pre* mutants. While other single *pre* mutant did not show discernible differences in flowering and seed setting, a few early formed flowers in *pre6* failed to set seeds (Figure S3). Further, the weak sterility of *pre6* was dramatically exaggerated by additional loss-of-function of *PRE2* or *PRE5* genes (Figure 2A). The double mutants of *pre2pre6* and *pre5pre6* showed fertility of about 35% of fully mature seeds, whereas the fertility of *pre4pre6* was similar to that of *pre6*. In contrast, *pre2pre4*, *pre2pre5*, or *pre4pre5* mutants showed normal fertility, compared to wild type. These results implied that PRE6 plays a critical role, redundantly with PRE2 or PRE5, in the reproductive process. Supporting this, the *pre2pre5pre6* triple mutant showed very low fertility such that it was difficult to get any mature siliques. In the case of other triple mutants, the fecundity of *pre2pre4pre6* or *pre4pre5pre6* was comparable to that of *pre2pre6* or *pre5pre6* double mutants (Figure 2A), which is indicative of the marginal effect of the *pre4* mutation.

**Figure 3:**
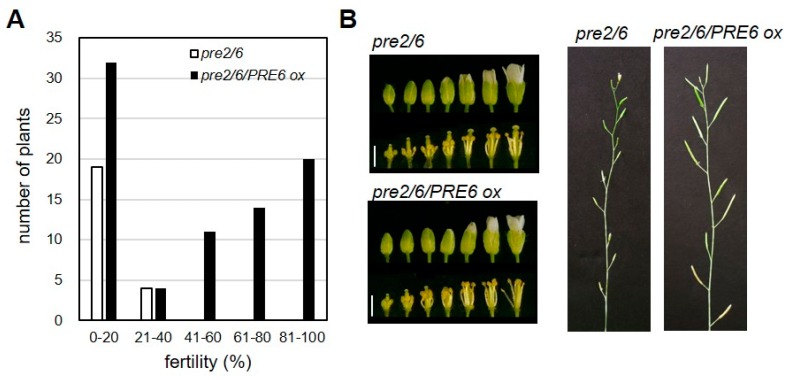
*PRE6* overexpression rescued the sterility of *pre2pre6*. The *pre2pre6* plants were transformed with Agrobacterium carrying the *PRE6* overexpressing vector. Full-length cDNA of *PRE6* was cloned into the plant overexpression vector. The primary transformants (T1) were selected and subjected to fertility analysis. (**A**) Fertility of T1 plants was scored by percentage of fully mature siliques per total flowers of the primary inflorescence. We analyzed 81 T1 plants of overexpressing *PRE6* in *pre2pre6* mutant background. (**B**) Left, representative flower morphology at various developmental stages. Scale bar, 1 mm. Right, representative primary inflorescences of *pre2pre6* and a transgenic line overexpressing *PRE6*.

To verify that the loss of *PRE* genes was responsible for the sterile phenotypes, we performed transgenic complementation by overexpressing *PRE6* or *PRE2* in the *pre2pre6* mutant background. The results showed that multiplmente independent lines of either *PRE6* or *PRE2* overexpression restored the reduced fertility of *pre2pre6* plants, demonstrating that the loss of function of these *PRE*s is responsible for the reduced fertility in the double mutant (Figure 3 and Figure S6).

Next, we examined the development of floral organs of the *pre2pre5pre6* mutant in detail to understand the cellular basis of the reduced fertility. The morphology of bud and style was indistinguishable between wild type and the triple mutant at various flower developmental stages (Figure 2B). On the other hand, the length of anther filament in the *pre2pre5pre6* was clearly shorter than that in wild type, suggesting that the reduced elongation of anther filament in the *pre2pre5pre6* might limit access of its pollens to stigma. Scanning electron microscopy (SEM) analysis revealed that the shorter anther filament of the *pre2pre5pre6* is mainly due to the reduced cell length of anther filament (Figure 2C). To test whether the pollens of the *pre2pre5pre6* mutant are functional for fertilization, we performed self-pollination assay with the *pre2pre5pre6* mutant by manual crossing. The self-pollinated mutant flowers could set fully mature seeds (Figure 2D), indicating that the reduced fertility in the *pre2pre5pre6* mutants may not be due to defective pollen development or anther dehiscence.

**Figure 4:**
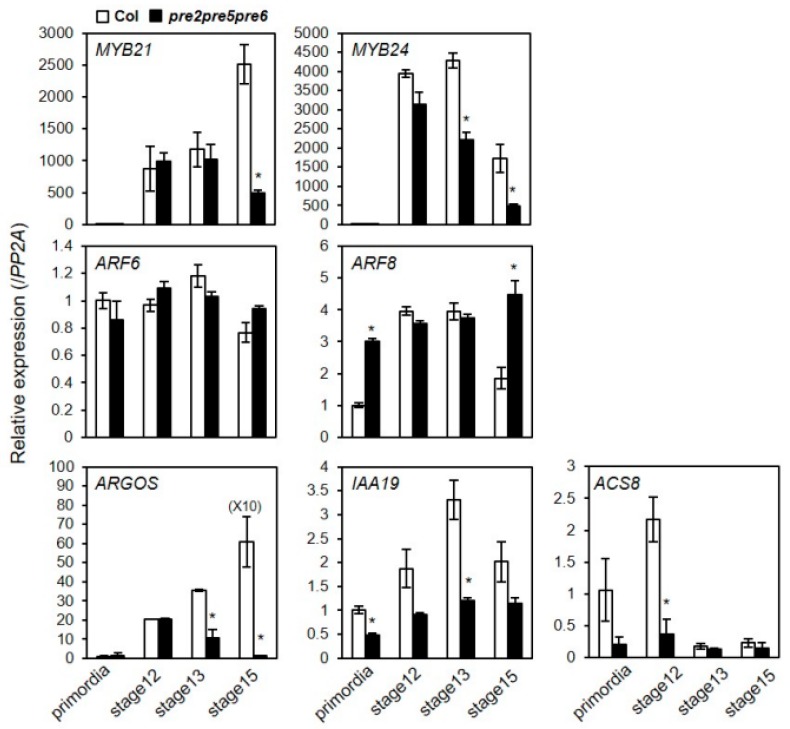
The expression of flower development-related genes was downregulated in *pre2pre5pre6* flowers. Total RNAs were extracted from flowers at indicated flower developmental stages and relative transcript levels were examined by quantitative RT-PCR analysis. The *PP2A* gene was analyzed as an internal control. Values are mean ± SD from three technical replicates. Significant difference between wild type (Col) and *pre2pre5pre6* is indicated with asterisk (*t*-test, * *p* < 0.01).

The sterility and floral phenotype of the *pre2pre5pre6* mutant were reminiscent of defects seen in mutants of transcriptional regulators, MYB21 or ARF6/ARF8 [11,31], which impair proper growth of anther filament. Previous expression analysis has suggested that *PRE* genes are auxin-inducible, being targeted by auxin-response factors including ARF5/ARF6/ARF8 [16,20,32,33]. In agreement, our RT-PCR analysis indicated that *PRE1*, *PRE2*, *PRE5*, and *PRE6* were downregulated in the *arf6arf8* double mutant or *myb21* mutant (Figure S7). Keeping in mind the hypothesis that PREs would act as a transcriptional regulator, we questioned whether the expression of those flower development-related genes or their target genes were altered in the *pre* mutant flowers. To address this question, we analyzed the expression of a selected set of genes including *MYB24*, *MYB21*, *ARF6*, *ARF8*, *ARGOS*, *IAA19*, and *ACS8* in the flowers at various developmental stages. The results showed that expression of *MYB24, ARGOS*, *IAA19*, and *ACS8* was downregulated in the *pre2pre5pre6* at stage 12 and 13, compared to wild type. In contrast, the transcript level of *MYB21* and *ARF6* was not altered in the *pre2pre5pre6*. In the case of *ARF8* expression, it was rather upregulated in the *pre2pre5pre6*, compared to wild type in the floral primordia and flower at stage 15 (Figure 4). Thus, these results implied that these PREs are involved in regulation of a subset of flower-developmental genes such as *ARGOS*, *ACS8*, *IAA19*, and *MYB24* acting presumably at the downstream of ARF6/ARF8-MYB21/MYB24 network.

### 2.3. Expression of PRE6 during Flower Development

**Figure 5:**
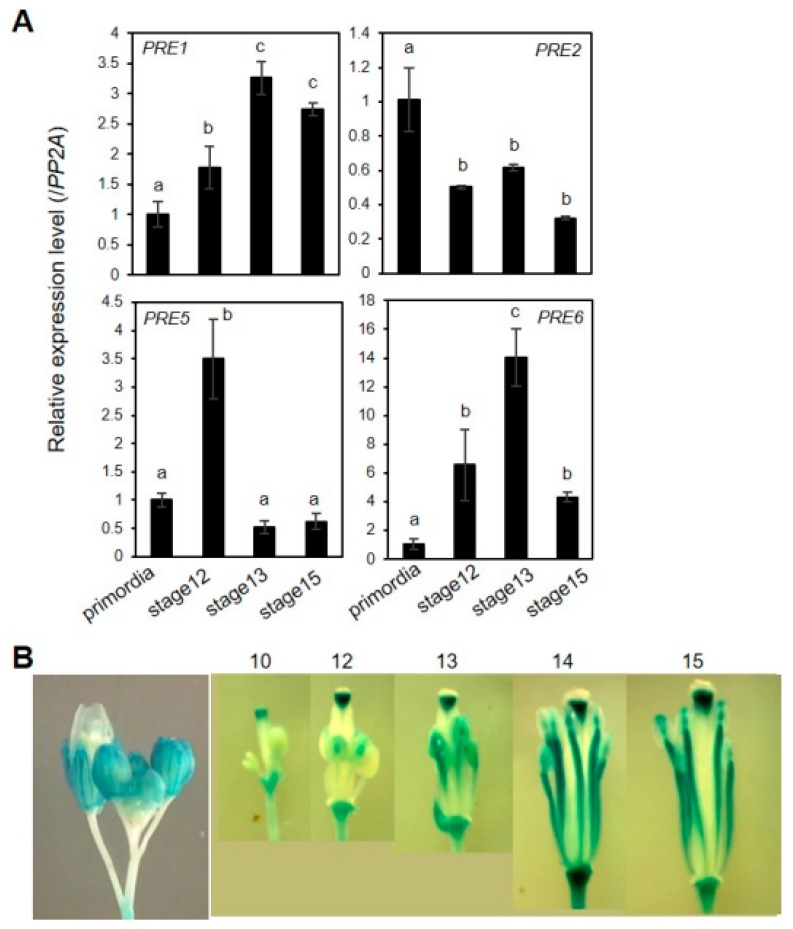
Expression of *PRE* genes during flower maturation. (**A**) Quantitative RT–PCR analyses of gene expression of *PREs* in developing flowers. Total RNAs were isolated from flowers at specific flower developmental stages. The data was represented as a relative value to primordia that was set to 1. The *PP2A* gene was used as an internal control. Values are mean ± SD from three technical replicates. Significant difference among flower development stages is indicated with letters (ANOVA, Tukey HSD test, *p* < 0.05). (**B**) *PRE6*pro: GUS analysis. *PRE6* promoter activity was monitored by GUS staining of transgenic plants expressing *PRE6*pro: *GUS* at indicated flower developmental stages.

We performed real-time qRT-PCR analysis to investigate the spatiotemporal expression pattern of *PRE*s during flower development in detail. The results showed that the expression of most of *PREs,* except *PRE2,* appear to correlate with the organ growth phase (Figure 5A). In the case of *PRE2*, its expression level was shown to be highest in the flower primordia, implying that *PRE2* might act at the very early stage of flower maturation. Given the prominent role of *PRE6* in the anther filament growth, we generated *PRE6*pro: *GUS* transgenic plants to analyze the spatial expression pattern of *PRE6* during flower development. The results of GUS staining showed that *PRE6* is expressed in various floral tissues, including sepals, a specific part of the style, just below the stigma, and the anther filament (Figure 5B). The expression of *PRE6* in anther filaments increased as filaments elongated, in accordance with the role of *PRE6* in cell elongation (Figure 5B). During vegetative growth, qRT-PCR analysis combined with promoter-GUS analysis indicated that the expression of *PRE6* increased in the actively growing, young leaves, while declined in mature leaves (Figure S8). Together, these results implied that the expression pattern of *PRE6* coincides largely with its functional aspects.

## 3. Discussion

The presence of a functionally redundant *PRE* gene family, comprised of six genes in *Arabidopsis thaliana*, raised an intriguing question about the *in planta* role of individual *PRE* genes during development. Although ectopic expression of each *PRE* gene induces very similar developmental alterations, including increased germination potential, early flowering, and elongated vegetative organ growth [18], the differential spatial expression pattern implied that PREs might play differential functions depending on the developmental contexts (Figure S2) [18]. Further, it has been well documented that expression of *PRE* genes showed differential inducibility upon various plant hormones or environmental signals [23,25].

In this study, we tried to identify the organ-specific role of PREs by performing reverse genetic analysis with multiple high order loss-of-function mutants of *PREs*. Our results not only recapitulated the role of PREs during vegetative growth based on artificial microRNA-mediated knock-down of multiple PREs [23,25], but also revealed their unequally redundant roles for floral organ growth, contributing to successful reproduction (See below). The single *pre* loss-of-function mutants and most of the double mutants we tested including *pre2pre4*, *pre2pre5*, *pre4pre5*, *pre4pre6*, and *pre5pre6* did not show altered organ growth, which is suggestive of functional compensation by other PREs. The reduced growth of hypocotyl and petiole was evident only in the mutants including both the *pre2* and *pre6*; *pre2pre6*, *pre2pre4pre6*, *pre2pre5pre6*, and *pre2pre4pre5pre6*. In contrast, the high order *pre* mutants, *pre2pre4*, *pre2pre5*, *pre4pre5*, *pre5pre6*, *pre4pre6*, and *pre4pre5pre6* did not exhibit any alteration in vegetative growth. Thus, these results indicated both PRE2 and PRE6 play dominant roles, in a redundant way, for the regulation of vegetative development, including hypocotyl and petiole/leaf growth.

While previous expression analysis showed that *PRE*s are expressed throughout floral development, regulated by floral homeotic genes (AP3/PI) or floral developmental regulators, ARF6/ARF8 [16,19], functional roles of *PRE*s have not been assessed during flower development. Here, we report the role of *PRE*s as a regulator of anther filament growth. Loss of *PRE6* caused slightly reduced fertility, which was exacerbated by the additional loss of *PRE2* or *PRE5*. In support of the roles of *PRE*s in fertility, transgenic expression of *PRE2* or *PRE6* complement the reduced fertility of the *pre2pre6* mutant (Figure 3 and Figure S6). In contrast, *pre2pre5* and *pre4pre6* did not show reduced fertility phenotype. Thus, it is conceivable that PRE6 plays a dominant role in the regulation of growth of anther filament, partly compensated by PRE2 and PRE5. In agreement with functional redundancy among *PRE2*, *PRE5*, and *PRE6*, the *pre2pre5pre6* mutant could rarely set seeds without manual self-crossing. In accordance with the roles of *PRE* for growth of anther filament, publicly available transcriptome variation analysis showed that these *PREs* (*PRE2*, *PRE5,* and *PRE6*) are strongly expressed in anther filaments during floral development (Figure S2) [34]. Since other *PRE*s, such as *PRE1* are also expressed in other floral organs, including sepals, petals, and carpels, it is likely that these PREs may be functional to support normal growth in other floral organs in the *pre2pre5pre6* mutant. Further studies with higher order *pre* mutants using a null allele of *PRE1*, *PRE3* or *PRE4* will shed light on additional roles in flower maturation.

SEM analysis indicated that the sterility of the *pre2pre5pre6* mutant was mainly ascribed to reduced cell elongation of anther filament (Figure 2). However, it is not yet known how PREs control cell elongation of anther filament. Previous microarray analysis of *arf6arf8* and *myb21* revealed that *PRE1* and *PRE2* are down-regulated at stage 12 and 13 [16,31]. This was confirmed by our RT-PCR analysis (Figure S7), suggesting that PREs would act downstream of the ARF6/ARF8-MYB21/MYB24 transcriptional regulatory network for flower maturation. In line with, we found that the expression of *ARGOS*, *IAA19,* and *ACS8* was significantly reduced in the *pre2pre5pre6* at stages when anther filament grows vigorously. As these genes are auxin-inducible and required for flower maturation [35,36], it is tempting to speculate that the sterility of the *pre* mutants might entail altered auxin responses and auxin-responsive growth. In line with the hypothesis, *PRE* has also been implicated in auxin-dependent responses such as hypocotyl growth and root development [20,32,33]. Thus, it is conceivable that a gene regulatory network for flower maturation involves a circuit of transcriptional regulators including ARF-PRE-IBH1-HBI/ACEs, which has been proposed for a transcriptional network for cell elongation during seedling establishment [32]. In agreement, it has been reported that constitutive overexpression of *IBH1* caused smaller flowers, with smaller cell length, while transgenic *ACE1*-overexpressing plants produced bigger flowers [26].

Our results showed that the effect of the *pre5* mutation is more prominent in the reproductive stage, while that of *pre2* and *pre6* was evident during both vegetative growth and reproductive development (Figure 1 and Figure 2). In the case of the *pre4* mutant, it marginally affected developmental aspects examined in this study. The absence of phenotypic effects could be due to its hypomorphic nature (Figure S3). Indeed, we did not observe a pale green sepal phenotype in the *pre4* mutant, which is a representative visible phenotype of a null allele of *PRE4* [19]. Alternatively, *PRE4* may play primary roles in certain environmental and developmental contexts not covered in this study. Considering that *PRE4*, along with other *PRE*s such as *PRE3,* is abundantly expressed in the root (Figure S2), it would be interesting to test root growth phenotypes of high order *pre* mutants under various environmental conditions. Nonetheless, our results suggest that each *PRE* gene plays an unequally redundant role. For the time being, the molecular basis of the unequal redundancy has not been clearly addressed. In the case of *PRE*s, the expression pattern appears to correlate largely to its biological functions. Several growth-promoting stimuli such as GA, BR, auxin, shade, high temperature were shown to entail upregulation of some, if not all, of *PRE* genes differentially [20,23,25,32,33]. In line with these findings, we also observed prominent expression of *PRE6* during leaf growth as well as floral organs, including anther filament (Figure 5 and Figure S8). Assuming that PREs function by forming a heterodimer with other HLH/bHLH proteins [22,23,37], it also cannot be ruled out that each PRE might have a differential affinity to its binding proteins. Indeed, IBH1 was reported to interact with PRE1, PRE3, PRE4, and PRE5, but not with PRE2 and PRE6 [26], whereas AIF1, another PRE-interacting protein, was shown to interact with PRE3, but not with PRE1 [21,27]. In contrast, PAR1 and PAR2, another class of PRE-interacting HLH proteins [29], could interact with all of the PRE proteins (Figure S1). As such, the differential expression of PRE-interacting proteins might also contribute to functional specificity among PREs. Thus, further study toward systems-wide understanding will determine the molecular basis of unequal redundancy among *PRE* genes under various environmental and developmental contexts.

## 4. Materials and Methods

### 4.1. Plant Materials

*Arabidopsis thaliana* ecotype Columbia (Col-0) was used as a wildtype in this study. The mutants of *myb21-5* (*myb21*) and *arf6-2arf8-3* (*arf6arf8*) have been previously described [16], and were obtained from Dr. J Reed (University of North Carolina-Chapel Hill, USA). T-DNA insertional mutants of *PRE* genes were obtained from the Arabidopsis Biological Resource Center (https://abrc.osu.edu/) and the Nottingham Arabidopsis Stock Centre (http://arabidopsis.info/); N122628 (*pre2*), SALK_098908C *(pre4*), SALK_013011 (*pre5*), and SALK_048383 (*pre6*). To make double *pre* mutants, the single T-DNA insertional mutants were crossed with each other. Triple and quadruple mutants of *pre2pre5pre6*, *pre2pre4pre6*, *pre4pre5pre6* and *pre2pre4pre5pre6* mutants were generated by crossing of *pre2pre5* with *pre5pre6* or *pre2pre4* with *pre5pre6.* The genotypes of high order *pre* mutants were confirmed by genotyping PCR and RT-PCR analysis with gene-specific primer pairs (Table S2).

### 4.2. Phenotypic Analysis

Unless otherwise stated, seeds were surface sterilized, kept at 4 °C for 3 days, and plated on the half strength of MS medium (Duchefa, Haarlem, Netherlands) supplemented with 0.5% sucrose and 0.7% phytoagar. After 7 d, they were transferred to soil (Sunshine Professional Growing Mix #5) for further growth under long days (16 h light/8 h dark) at 21 to 23 °C. For hypocotyl measurement, seedlings were grown either under short-day (8 h light/16 h dark) W light (15 μW cm^−2^) or in the dark for 4 days. For measurement of petiole and leaf length, plants were grown under long-day light conditions (16 h light/8 h dark). Petiole and leaf lengths were measured from the 7th leaf of 3 weeks old plants. The floral bud and dissected floral organs at different flower development stages, denoted by Smyth et al. [38], were taken and scanned using a flatbed scanner to measure organ lengths. The measurement of organ length was performed using Image J software (https://imagej.nih.gov/ij/). Flowering time was measured by rosette leaf numbers when the first clear appearance of a flower petal that was visible. Fertility was denoted by the percentage of fertile siliques, bearing seeds, among flowers formed on the primary inflorescence at 40 days after growth. For scanning electron microscopy, petioles and flowers were fixed and processed as described [31]. Briefly, petioles and flowers were fixed with modified Karnovsky’s fixative (2% paraformaldehyde, 2% glutaraldehyde in 0.05 M sodium cacodylate buffer, pH 7.2) for 2 h at 4 °C. After washing with 0.05 M sodium cacodylate buffer (pH 7.2), the sample was fixed further in 1% osmium tetroxide for 2 h at 4 °C. Washed sample was dehydrated with a series of Ethanol from 30% to 100%. After critical point drying (Balzer’s critical point drier), the samples were mounted on aluminum stubs and sputter-coated (SC7620; Quorum) with gold for SEM imaging. The specimens were imaged in a Field-Emission Scanning Electron Microscope (ZEISS, Oberkochen, Germany).

### 4.3. Transgenic Complementation of pre2pre6

Full-length cDNA of *PRE2* or *PRE6* was amplified with specific primer sets designed with the overhang of restriction enzyme site, KpnI and XhoI for *PRE2* and BamHI and XhoI for *PRE6* respectively; 5′-GGC***GGTACC***CAATGTCTTCTAGCAGAAGG-3′ and 5′-GGC***CTCGAG***GTTCCATTAATCAAGCTCCT-3′ for *PRE2* and 5′- ***GGATCC***AGATGTCTAGCAGAAGATCATC-3′ and 5′-***CTCGAG***ATATAATTAAGCAAGCTCCTAA-3′ for *PRE6*. The amplified PCR products were subcloned into the pGEM-T easy vector (Promega, Madison, USA). After verification no sequence error, the vector containing *PRE2* or *PRE6* was digested with KpnI/XhoI or BamHI/XhoI for *PRE2* or *PRE6,* respectively. The digest was gel-eluted and ligated with pENTR1A digested with the same restriction enzyme set (Invitrogen, Carlsbad, CA, USA). The cloned entry clone was recombined into a gateway-compatible plant expression vector, pH2GW7 using LR clonase [39], respectively. The resulting construct was transformed into *Agrobacterium tumefaciens* strain GV3101, which was used for the transformation of the *pre2pre6* plants by the floral dipping method [40]. More than 30 transgenic lines with T-DNA insertions were isolated and used for physiological analysis.

### 4.4. Expression Analysis

Total RNA from different tissues was extracted with a Spectrum total RNA extraction kit (Sigma-Aldrich, St. Louis, MO, USA) according to the manufacturer’s instruction. One µg of RNA was reverse transcribed by using RevertAid™ M-MuLV Reverse Transcriptase (Fermentas, Waltham, MA, USA). The 10-fold diluted cDNA was subject to RT-PCR analysis or qRT-PCR analysis using primers listed in Table S2. The qRT-PCR analysis was performed with an Eco™ Real-Time PCR System (Illumina, San Diego, CA, USA) using a QuantiMix SYBRT kit (Philekorea, Seoul, South Korea). The reactions were performed in triplicate for each run. The relative expression level of each gene was determined after being normalized to that of *PP2A* (At1g13320). The comparative 2^–ΔΔ*C*t^ method was used to evaluate the relative quantities of each amplified product in the samples, following the procedure as described [41].

### 4.5. Yeast Two-hybrid Analysis

Yeast two-hybrid (Y2H) assays were performed following the procedure as described in [42]. The *PRE* cDNAs were cloned into the pGilda vector (Clontech, Mountain View, USA), except for *PRE6* which is subcloned to gGilda by LR recombination to generate a LexA-DB fused construct. *PRE1*-*PRE5* cDNA was amplified with specific primer set for pGilda cloning. All primer sets include EcoRI and XhoI restriction enzyme site for cloning into the pGilda vector. The amplified fragments were cloned into a pGEM-T easy vector. After verification of no sequence error, the digested *PRE* cDNA fragments were ligated with pGilda vector. The full-length of the *PAR1* and *PAR2* cDNAs were cloned into pYesTrp2 vector (Invitrogen, Carlsbad, CA, USA) to produce the B42-AD fused protein. The specific primer sets for *PAR1* and *PAR2* include HindIII and XhoI restriction enzyme sites for cloning into the pYesTrp2 vector. After cloning into a pGEM-T easy vector, the digested *PAR1* or *PAR2* cDNA fragments were subcloned into the pYesTrp2 vector. The primer set used for cloning is listed in Table S2. All the constructs were sequence verified. The construct pairs were transformed into the yeast, *Saccharomyces cerevisiae* strain EGY48, and cultured at 30 °C. Successful yeast transformants were cultured in liquid SD minimal medium (-Ura/-His/-Trp) at 230 rpm/30 °C for 24 h so that it reached a final concentration equivalent to OD600, 1.2 to 1.5. Then, 1 mL of yeast strain was centrifuged and resuspended in 0.3 mL of distilled water. Ten microliters of the suspension cells were plated on SD/Gal/Raf (-Ura/-His/-Trp/-Leu) induction medium (MP Biomedicals, Santa Ana, CA, USA). Y2H images were taken 3 d after incubation at 30 °C.

### 4.6. Promoter Activity Analysis

A 1.2 kb DNA fragment containing the 5′ upstream region of *PRE6* was amplified with primers (5′-GGATCCTGCCATTTTAAACCCCACTT-3′ and 5′-CGATCGGTGTTGAAGAGGGAGGAGCA-3′). The amplified fragment was cloned into a pGEM-T Easy vector, after verification of no sequence error, then subcloned into the pCAMBIA1381 vector. The resulting recombinant plasmid was transformed into the *Agrobacterium tumefaciens* strain GV3101, and introduced into wild type plants using the floral dipping method. Histochemical analysis of GUS activity in Arabidopsis tissues was done as described by Jefferson et al. (1987). The stained tissues were post-fixed in FAA (4% formaldehyde (*v/v*), 5% acetic acid (*v/v*), and 50% ethanol (*v/v*)) and cleared in 70% ethanol before being photographed. Whole-mount tissue samples were viewed with Axioplan microscopes (Carl Zeiss). Digital images were captured using a Spot II camera (Diagnostic Instruments Inc, Starling Height, MI, USA).

## 5. Conclusions

In summary, our study with loss-of-function *pre* mutants not only confirmed the regulatory function of *PRE*s in cell elongation during vegetative development but also revealed the role of *PRE*s in cell elongation of anther filament, which is critical for successful pollination. Taking together with the results from gain-of-function studies, we suggest that PREs may constitute versatile growth-regulatory units in various developmental contexts. As exemplified in the previous report that altered expression of a *PRE* ortholog, *Style2.1*, is associated with the evolution of self-pollination in cultivated tomato species [3], we propose that manipulation of *PRE* expression using tissue-specific promoters is a promising way to manipulate self-pollination behavior of flowering plants, an agronomically important trait.

## Supplementary Materials

Supplementary Materials can be found at http://www.mdpi.com/1422-0067/20/4/869/s1.

## Abbreviations

PRE	PACLOBUTRAZOL RESISTANCE
WT	Wild type
GA	Gibberellin
BR	Brassinosteroid
JA	Jasmonic acid
ARF	AUXIN RESPONSE FACTOR
qRT-PCR	Quantitative real-time PCR
HLH	Helix loop helix

## References

[1] Karron J.D., Jackson R.T., Thumser N.N., Schlicht S.L. (1997). Outcrossing rates of individual Mimulus ringens genets are correlated with anther–stigma separation. Heredity.

[2] Motten A.F., Stone J.L. (2000). Heritability of stigma position and the effect of stigma-anther separation on outcrossing in a predominantly self-fertilizing weed, Datura stramonium (Solanaceae). Am. J. Bot..

[3] Chen K.Y., Cong B., Wing R., Vrebalov J., Tanksley S.D. (2007). Changes in regulation of a transcription factor lead to autogamy in cultivated tomatoes. Science.

[4] Cecchetti V., Altamura M.M., Falasca G., Costantino P., Cardarelli M. (2008). Auxin regulates Arabidopsis anther dehiscence, pollen maturation, and filament elongation. Plant Cell.

[5] Huang H., Gao H., Liu B., Qi T., Tong J., Xiao L., Xie D., Song S. (2017). Arabidopsis MYB24 Regulates Jasmonate-Mediated Stamen Development. Front Plant Sci..

[6] Park J.H., Halitschke R., Kim H.B., Baldwin I.T., Feldmann K.A., Feyereisen R. (2002). A knock-out mutation in allene oxide synthase results in male sterility and defective wound signal transduction in Arabidopsis due to a block in jasmonic acid biosynthesis. Plant J..

[7] Singh M., Gupta A., Singh D., Khurana J.P., Laxmi A. (2017). Arabidopsis RSS1 Mediates Cross-Talk Between Glucose and Light Signaling During Hypocotyl Elongation Growth. Sci. Rep..

[8] Cheng H., Qin L., Lee S., Fu X., Richards D.E., Cao D., Luo D., Harberd N.P., Peng J. (2004). Gibberellin regulates Arabidopsis floral development via suppression of DELLA protein function. Development.

[9] Ishiguro S., Kawai-Oda A., Ueda J., Nishida I., Okada K. (2001). The DEFECTIVE IN ANTHER DEHISCIENCE gene encodes a novel phospholipase A1 catalyzing the initial step of jasmonic acid biosynthesis, which synchronizes pollen maturation, anther dehiscence, and flower opening in Arabidopsis. Plant Cell.

[10] Koornneef M., van der Veen J.H. (1980). Induction and analysis of gibberellin sensitive mutants in Arabidopsis thaliana (L.) heynh. Theor. Appl. Genet..

[11] Mandaokar A., Thines B., Shin B., Lange B.M., Choi G., Koo Y.J., Yoo Y.J., Choi Y.D., Choi G., Browse J. (2006). Transcriptional regulators of stamen development in Arabidopsis identified by transcriptional profiling. Plant J..

[12] Sanders P.M., Lee P.Y., Biesgen C., Boone J.D., Beals T.P., Weiler E.W., Goldberg R.B. (2000). The arabidopsis DELAYED DEHISCENCE1 gene encodes an enzyme in the jasmonic acid synthesis pathway. Plant Cell.

[13] Stintzi A., Browse J. (2000). The Arabidopsis male-sterile mutant, opr3, lacks the 12-oxophytodienoic acid reductase required for jasmonate synthesis. Proc. Natl. Acad. Sci. USA.

[14] Ye Q., Zhu W., Li L., Zhang S., Yin Y., Ma H., Wang X. (2010). Brassinosteroids control male fertility by regulating the expression of key genes involved in Arabidopsis anther and pollen development. Proc. Natl. Acad. Sci. USA.

[15] Cheng H., Song S., Xiao L., Soo H.M., Cheng Z., Xie D., Peng J. (2009). Gibberellin acts through jasmonate to control the expression of MYB21, MYB24, and MYB57 to promote stamen filament growth in Arabidopsis. PLoS Genet..

[16] Reeves P.H., Ellis C.M., Ploense S.E., Wu M.F., Yadav V., Tholl D., Chetelat A., Haupt I., Kennerley B.J., Hodgens C. (2012). A regulatory network for coordinated flower maturation. PLoS Genet..

[17] Hyun Y., Lee I. (2006). KIDARI, encoding a non-DNA Binding bHLH protein, represses light signal transduction in Arabidopsis thaliana. Plant Mol. Biol..

[18] Lee S., Lee S., Yang K.Y., Kim Y.M., Park S.Y., Kim S.Y., Soh M.S. (2006). Overexpression of PRE1 and its homologous genes activates Gibberellin-dependent responses in Arabidopsis thaliana. Plant Cell Physiol..

[19] Mara C.D., Huang T., Irish V.F. (2010). The Arabidopsis floral homeotic proteins APETALA3 and PISTILLATA negatively regulate the BANQUO genes implicated in light signaling. Plant Cell.

[20] Schlereth A., Moller B., Liu W., Kientz M., Flipse J., Rademacher E.H., Schmid M., Jurgens G., Weijers D. (2010). MONOPTEROS controls embryonic root initiation by regulating a mobile transcription factor. Nature.

[21] Wang H., Zhu Y., Fujioka S., Asami T., Li J., Li J. (2009). Regulation of Arabidopsis brassinosteroid signaling by atypical basic helix-loop-helix proteins. Plant Cell.

[22] Zhang L.Y., Bai M.Y., Wu J., Zhu J.Y., Wang H., Zhang Z., Wang W., Sun Y., Zhao J., Sun X. (2009). Antagonistic HLH/bHLH transcription factors mediate brassinosteroid regulation of cell elongation and plant development in rice and Arabidopsis. Plant Cell.

[23] Bai M.Y., Fan M., Oh E., Wang Z.Y. (2012). A triple helix-loop-helix/basic helix-loop-helix cascade controls cell elongation downstream of multiple hormonal and environmental signaling pathways in Arabidopsis. Plant Cell.

[24] Chapman E.J., Greenham K., Castillejo C., Sartor R., Bialy A., Sun T.P., Estelle M. (2012). Hypocotyl transcriptome reveals auxin regulation of growth-promoting genes through GA-dependent and -independent pathways. PLoS ONE.

[25] Oh E., Zhu J.Y., Wang Z.Y. (2012). Interaction between BZR1 and PIF4 integrates brassinosteroid and environmental responses. Nat Cell Biol.

[26] Ikeda M., Fujiwara S., Mitsuda N., Ohme-Takagi M. (2012). A triantagonistic basic helix-loop-helix system regulates cell elongation in Arabidopsis. Plant Cell.

[27] Ikeda M., Mitsuda N., Ohme-Takagi M. (2013). ATBS1 INTERACTING FACTORs negatively regulate Arabidopsis cell elongation in the triantagonistic bHLH system. Plant Signal Behav..

[28] De Rybel B., van den Berg W., Lokerse A., Liao C.Y., van Mourik H., Moller B., Peris C.L., Weijers D. (2011). A versatile set of ligation-independent cloning vectors for functional studies in plants. Plant. Physiol..

[29] Hao Y., Oh E., Choi G., Liang Z., Wang Z.Y. (2012). Interactions between HLH and bHLH factors modulate light-regulated plant development. Mol. Plant..

[30] Alonso-Blanco C., Andrade J., Becker C., Bemm F., Bergelson J., Borgwardt K.M., Cao J., Chae E., Dezwaan T.M., Ding W. (2016). 1,135 Genomes Reveal the Global Pattern of Polymorphism in Arabidopsis thaliana. Cell.

[31] Nagpal P., Ellis C.M., Weber H., Ploense S.E., Barkawi L.S., Guilfoyle T.J., Hagen G., Alonso J.M., Cohen J.D., Farmer E.E. (2005). Auxin response factors ARF6 and ARF8 promote jasmonic acid production and flower maturation. Development.

[32] Oh E., Zhu J.Y., Bai M.Y., Arenhart R.A., Sun Y., Wang Z.Y. (2014). Cell elongation is regulated through a central circuit of interacting transcription factors in the Arabidopsis hypocotyl. Elife.

[33] Zheng K., Wang Y., Zhang N., Jia Q., Wang X., Hou C., Chen J.G., Wang S. (2017). Involvement of PACLOBUTRAZOL RESISTANCE6/KIDARI, an Atypical bHLH Transcription Factor, in Auxin Responses in Arabidopsis. Front. Plant. Sci..

[34] Klepikova A.V., Kasianov A.S., Gerasimov E.S., Logacheva M.D., Penin A.A. (2016). A high resolution map of the Arabidopsis thaliana developmental transcriptome based on RNA-seq profiling. Plant. J..

[35] Okushima Y., Mitina I., Quach H.L., Theologis A. (2005). AUXIN RESPONSE FACTOR 2 (ARF2): A pleiotropic developmental regulator. Plant. J..

[36] Tashiro S., Tian C.E., Watahiki M.K., Yamamoto K.T. (2009). Changes in growth kinetics of stamen filaments cause inefficient pollination in massugu2, an auxin insensitive, dominant mutant of Arabidopsis thaliana. Physiol. Plant..

[37] Zhiponova M.K., Morohashi K., Vanhoutte I., Machemer-Noonan K., Revalska M., Van Montagu M., Grotewold E., Russinova E. (2014). Helix-loop-helix/basic helix-loop-helix transcription factor network represses cell elongation in Arabidopsis through an apparent incoherent feed-forward loop. Proc. Natl. Acad. Sci. USA.

[38] Smyth D.R., Bowman J.L., Meyerowitz E.M. (1990). Early flower development in Arabidopsis. Plant. Cell.

[39] Karimi M., Inze D., Depicker A. (2002). GATEWAY vectors for Agrobacterium-mediated plant transformation. Trends Plant. Sci..

[40] Clough S.J., Bent A.F. (1998). Floral dip: A simplified method for Agrobacterium-mediated transformation of Arabidopsis thaliana. Plant. J..

[41] Livak K.J., Schmittgen T.D. (2001). Analysis of relative gene expression data using real-time quantitative PCR and the 2^−ΔΔ*C*T^ Method. Methods.

[42] Choi H., Hong J., Ha J., Kang J., Kim S.Y. (2000). ABFs, a family of ABA-responsive element binding factors. J. Biol. Chem..

