# Supplementary material for: PACLOBUTRAZOL-RESISTANCE Gene Family Regulates Floral Organ Growth with Unequal Genetic Redundancy in Arabidopsis thaliana
Source: Int J Mol Sci. 2019 Feb 17;20(4):869. doi: 10.3390/ijms20040869 (PMC6412927; doi:10.3390/ijms20040869)
Supplement: Supplementary file 1 [file ijms-20-00869-s001.zip › Supplementary Figures.pptx]

## Slide 1
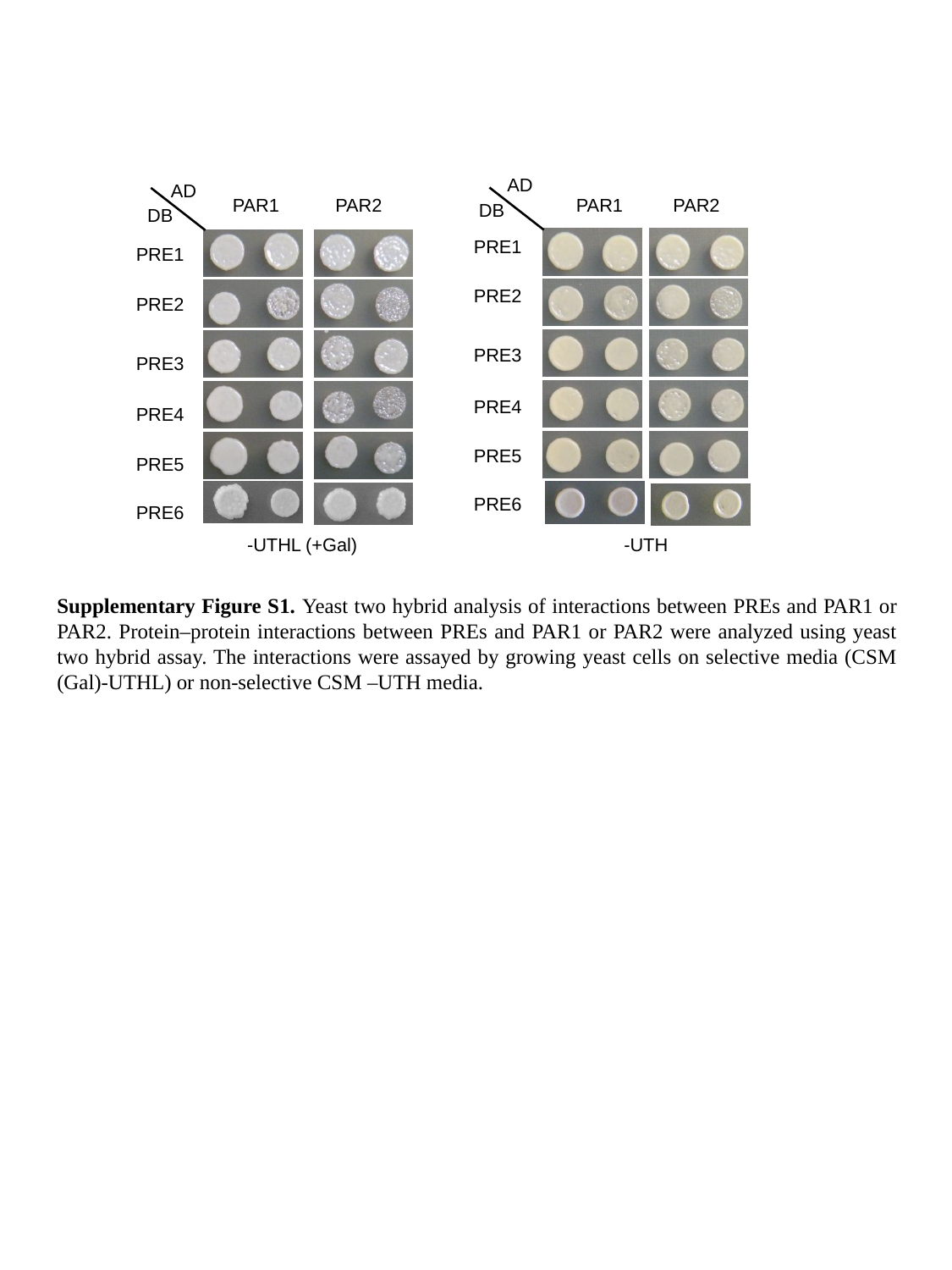

AD
DB
 AD
PAR1
PAR2
PAR1
PAR2
DB
PRE1
PRE1
PRE2
PRE2
PRE3
PRE3
PRE4
PRE4
PRE5
PRE5
PRE6
PRE6
-UTHL (+Gal)
-UTH
Supplementary Figure S1. Yeast two hybrid analysis of interactions between PREs and PAR1 or PAR2. Protein–protein interactions between PREs and PAR1 or PAR2 were analyzed using yeast two hybrid assay. The interactions were assayed by growing yeast cells on selective media (CSM (Gal)-UTHL) or non-selective CSM –UTH media.

## Slide 2
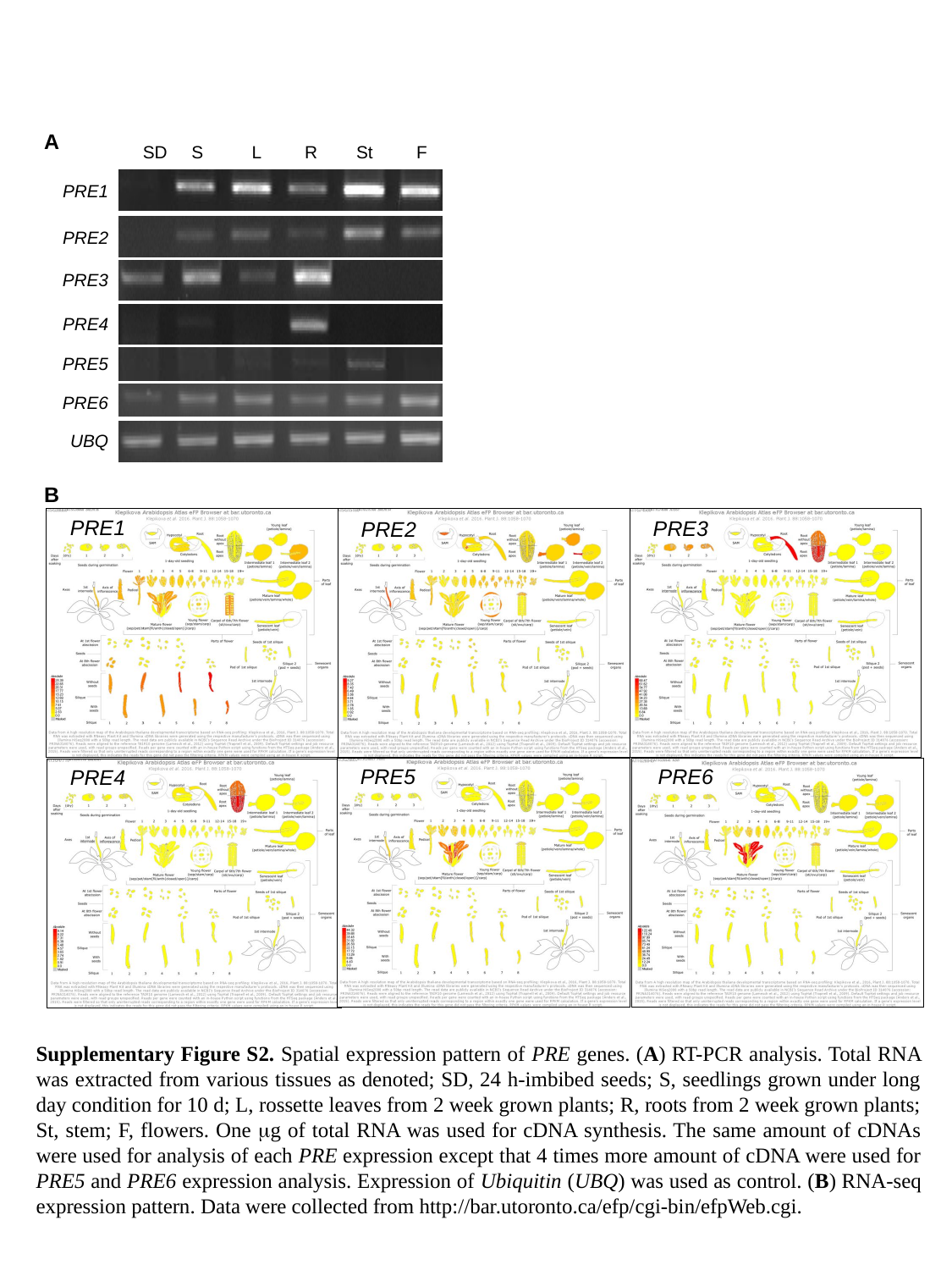

A
SD S L R St F
PRE1
PRE2
PRE3
PRE4
PRE5
PRE6
UBQ
B
PRE1
PRE3
PRE2
PRE5
PRE6
PRE4
Supplementary Figure S2. Spatial expression pattern of PRE genes. (A) RT-PCR analysis. Total RNA was extracted from various tissues as denoted; SD, 24 h-imbibed seeds; S, seedlings grown under long day condition for 10 d; L, rossette leaves from 2 week grown plants; R, roots from 2 week grown plants; St, stem; F, flowers. One μg of total RNA was used for cDNA synthesis. The same amount of cDNAs were used for analysis of each PRE expression except that 4 times more amount of cDNA were used for PRE5 and PRE6 expression analysis. Expression of Ubiquitin (UBQ) was used as control. (B) RNA-seq expression pattern. Data were collected from http://bar.utoronto.ca/efp/cgi-bin/efpWeb.cgi.

## Slide 3
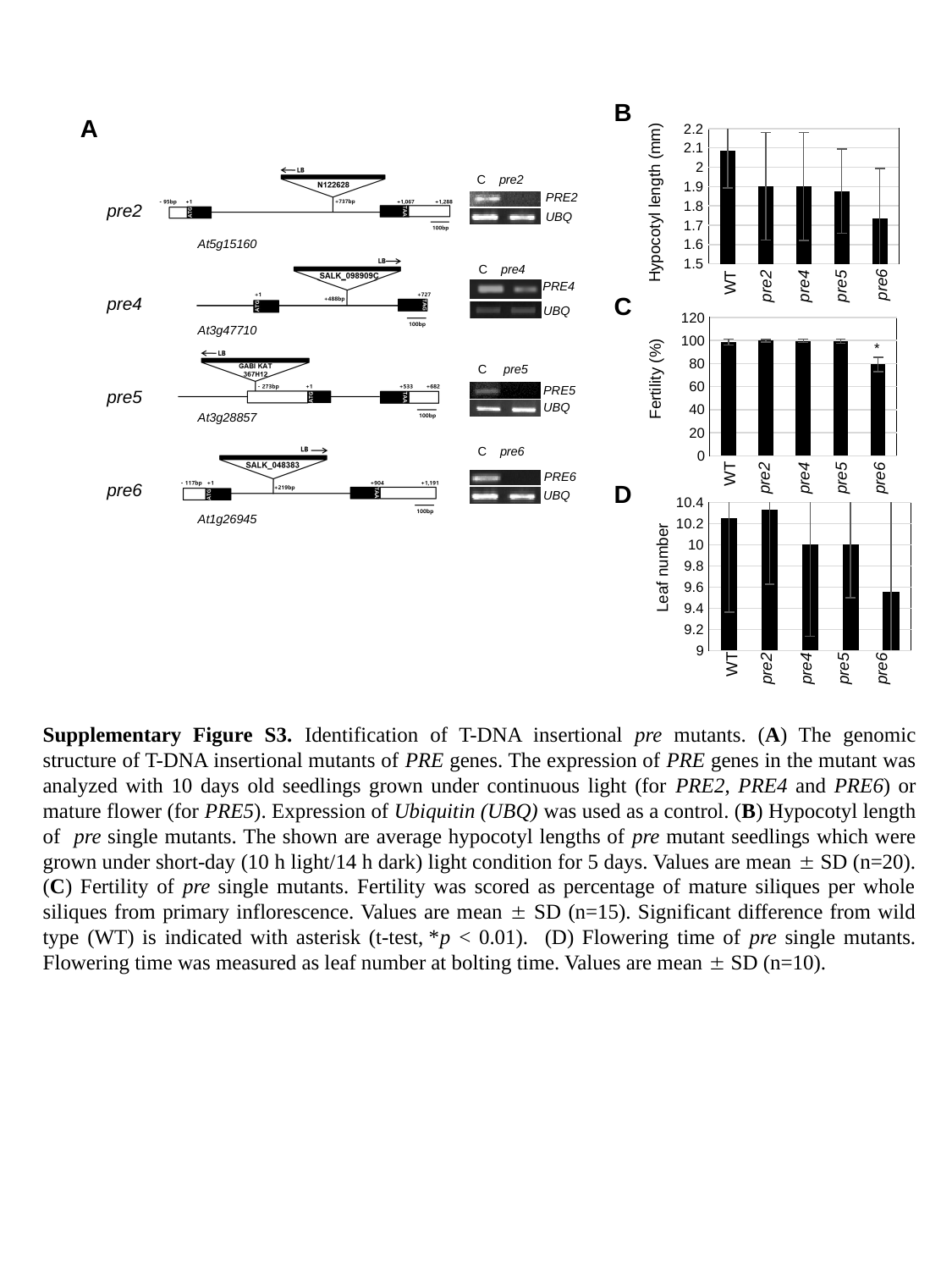

B
A
### Chart
| Category | |
|---|---|
| Col-0 | 2.083154779798187 |
| pre2KO | 1.901314901249807 |
| pre4KO | 1.900232038519685 |
| pre5KO | 1.8755087886109505 |
| pre6KO | 1.7363270117622676 |
C pre2
PRE2
UBQ
Hypocotyl length (mm)
pre2
At5g15160
C pre4
WT
pre6
pre2
pre4
pre5
PRE4
C
pre4
UBQ
### Chart
| Category | |
|---|---|
| Col-0 | 98.48129251700679 |
| pre2K | 99.68944099378882 |
| pre4K | 99.62406015037594 |
| pre5K | 99.31972789115646 |
| pre6K | 79.17721202193252 |
At3g47710
*
 C pre5
PRE5
UBQ
Fertility (%)
pre5
At3g28857
 C pre6
WT
pre2
pre4
pre5
pre6
PRE6
pre6
D
UBQ
### Chart
| Category | |
|---|---|
| Col-0 | 10.25 |
| pre2 | 10.333333333333334 |
| pre4 | 10.0 |
| pre5 | 10.0 |
| pre6 | 9.555555555555555 |At1g26945
Leaf number
WT
pre2
pre4
pre5
pre6
Supplementary Figure S3. Identification of T-DNA insertional pre mutants. (A) The genomic structure of T-DNA insertional mutants of PRE genes. The expression of PRE genes in the mutant was analyzed with 10 days old seedlings grown under continuous light (for PRE2, PRE4 and PRE6) or mature flower (for PRE5). Expression of Ubiquitin (UBQ) was used as a control. (B) Hypocotyl length of pre single mutants. The shown are average hypocotyl lengths of pre mutant seedlings which were grown under short-day (10 h light/14 h dark) light condition for 5 days. Values are mean ± SD (n=20). (C) Fertility of pre single mutants. Fertility was scored as percentage of mature siliques per whole siliques from primary inflorescence. Values are mean ± SD (n=15). Significant difference from wild type (WT) is indicated with asterisk (t-test, *p < 0.01). (D) Flowering time of pre single mutants. Flowering time was measured as leaf number at bolting time. Values are mean ± SD (n=10).

## Slide 4
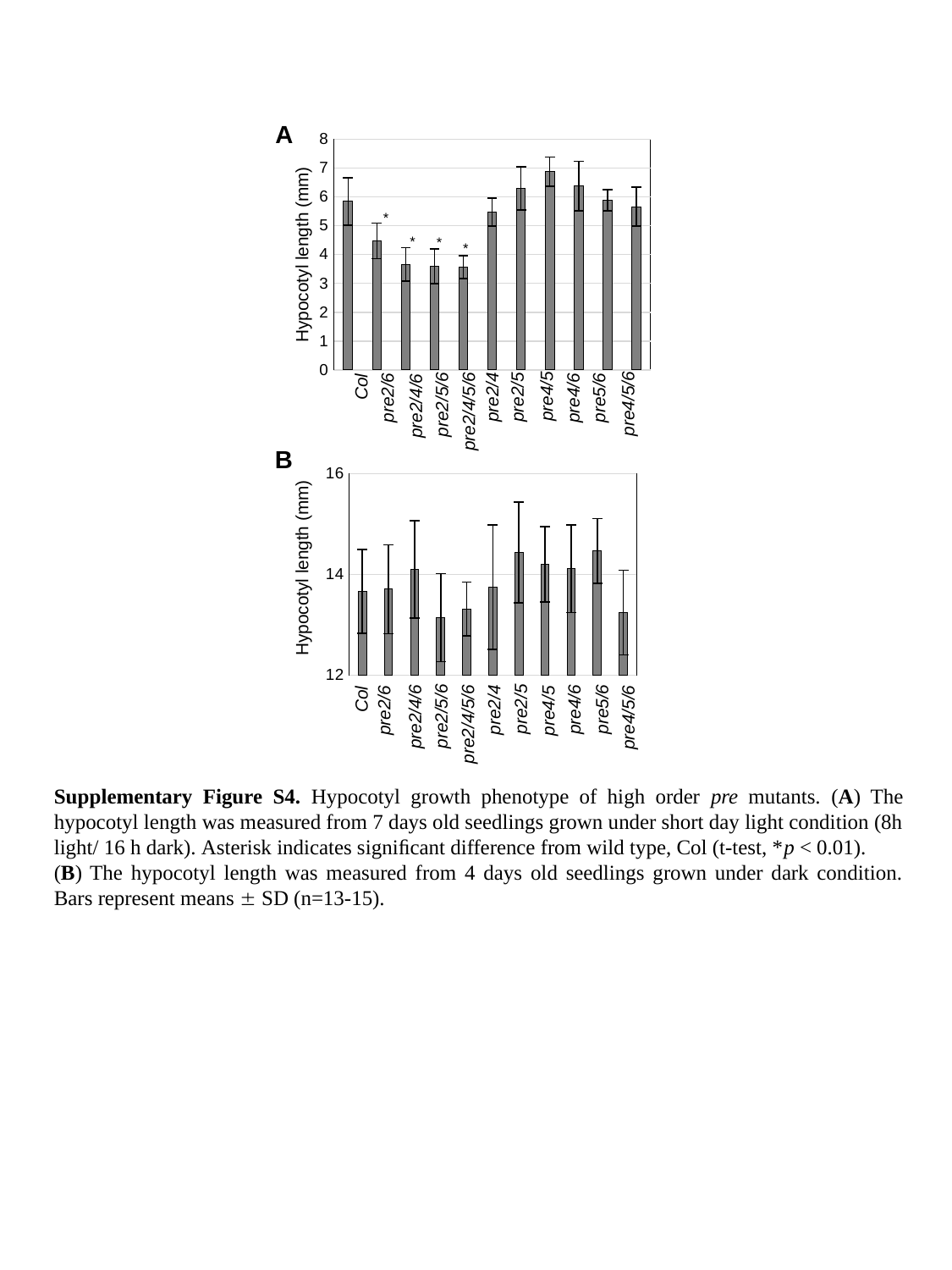

A
### Chart
| Category | AVG |
|---|---|
| Col-0 | 5.8447023119931485 |
| pre2/6 | 4.4688949886329645 |
| pre2/4/6 | 3.655886360754688 |
| pre2/5/6 | 3.5959048529279065 |
| pre2/4/5/6 | 3.556856048576899 |
| pre2/4 | 5.468485299338934 |
| pre2/5 | 6.291332720879399 |
| pre4/5 | 6.87559248545017 |
| pre4/6 | 6.374876936628842 |
| pre5/6 | 5.883565328676901 |
| pre4/5/6 | 5.658898407045576 |*
*
*
Hypocotyl length (mm)
*
Col
pre2/6
pre2/4
pre5/6
pre2/5
pre4/6
pre4/5
pre2/5/6
pre2/4/6
pre4/5/6
pre2/4/5/6
B
### Chart
| Category | |
|---|---|
| Col-0 | 13.659301526200435 |
| pre2/6 | 13.705940765640003 |
| pre2/4/6 | 14.10073625772632 |
| pre2/5/6 | 13.142594887683964 |
| pre2/4/5/6 | 13.310666846717863 |
| pre2/4 | 13.748280991531493 |
| pre2/5 | 14.437187667600636 |
| pre4/5 | 14.198077126386028 |
| pre4/6 | 14.112643050353725 |
| pre5/6 | 14.464489274350168 |
| pre4/5/6 | 13.24176685682547 |Col
pre2/6
pre5/6
pre2/4
pre2/5
pre4/6
pre2/5/6
pre2/4/6
pre4/5
pre4/5/6
pre2/4/5/6
Hypocotyl length (mm)
Supplementary Figure S4. Hypocotyl growth phenotype of high order pre mutants. (A) The hypocotyl length was measured from 7 days old seedlings grown under short day light condition (8h light/ 16 h dark). Asterisk indicates signiﬁcant difference from wild type, Col (t-test, *p < 0.01).
(B) The hypocotyl length was measured from 4 days old seedlings grown under dark condition. Bars represent means ± SD (n=13-15).

## Slide 5
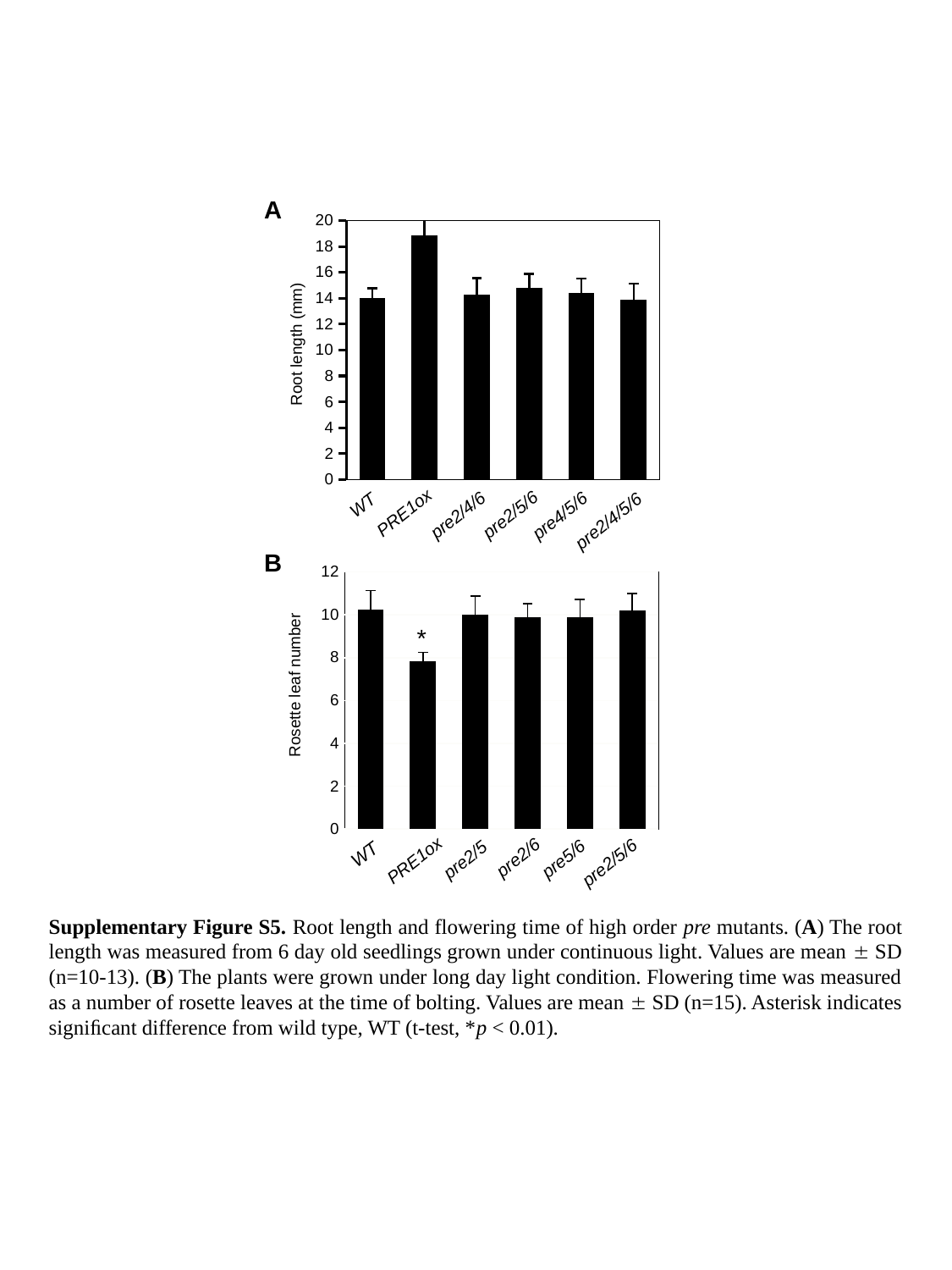

A
### Chart
| Category | |
|---|---|
| WT | 13.995085251143573 |
| PRE1ox | 18.826460670712997 |
| pre2/4/6 | 14.263940855980055 |
| pre2/5/6 | 14.83457544333286 |
| pre4/5/6 | 14.420264549909241 |
| pre2/4/5/6 | 13.913655380270411 |*
Root length (mm)
WT
PRE1ox
pre2/5/6
pre2/4/6
pre4/5/6
pre2/4/5/6
B
### Chart
| Category | |
|---|---|
| Col-0 | 10.25 |
| PRE1ox | 7.833333333333342 |
| pre2/5 | 10.0 |
| pre2/6 | 9.875000000000016 |
| pre5/6 | 9.875000000000016 |
| pre2/5/6 | 10.200000000000001 |Rosette leaf number
WT
pre2/6
pre5/6
pre2/5
PRE1ox
pre2/5/6
*
Supplementary Figure S5. Root length and flowering time of high order pre mutants. (A) The root length was measured from 6 day old seedlings grown under continuous light. Values are mean ± SD (n=10-13). (B) The plants were grown under long day light condition. Flowering time was measured as a number of rosette leaves at the time of bolting. Values are mean ± SD (n=15). Asterisk indicates signiﬁcant difference from wild type, WT (t-test, *p < 0.01).

## Slide 6
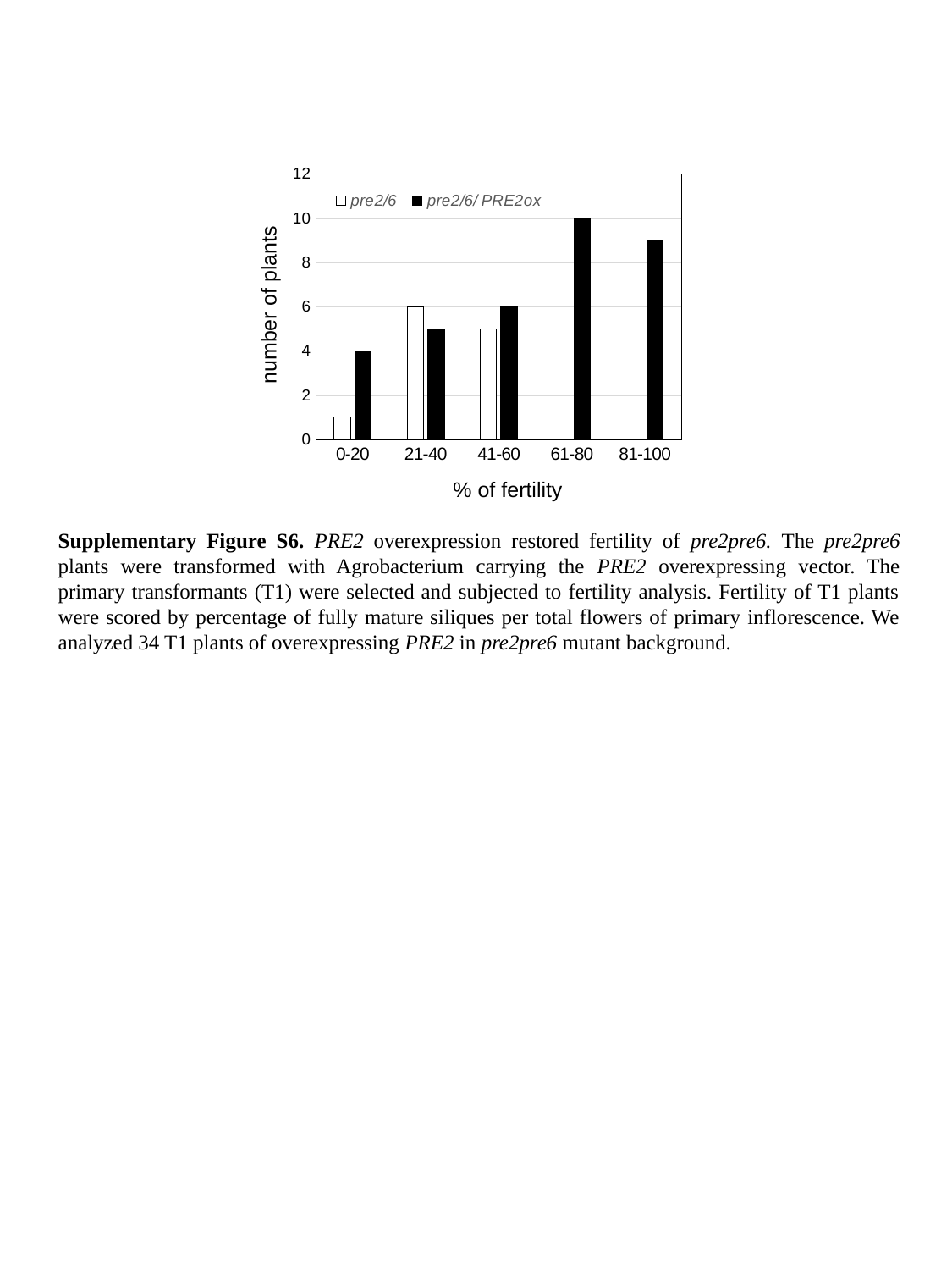

### Chart
| Category | pre2/6 | pre2/6/ PRE2ox |
|---|---|---|
| 0-20 | 1.0 | 4.0 |
| 21-40 | 6.0 | 5.0 |
| 41-60 | 5.0 | 6.0 |
| 61-80 | 0.0 | 10.0 |
| 81-100 | 0.0 | 9.0 |number of plants
% of fertility
Supplementary Figure S6. PRE2 overexpression restored fertility of pre2pre6. The pre2pre6 plants were transformed with Agrobacterium carrying the PRE2 overexpressing vector. The primary transformants (T1) were selected and subjected to fertility analysis. Fertility of T1 plants were scored by percentage of fully mature siliques per total flowers of primary inflorescence. We analyzed 34 T1 plants of overexpressing PRE2 in pre2pre6 mutant background.

## Slide 7
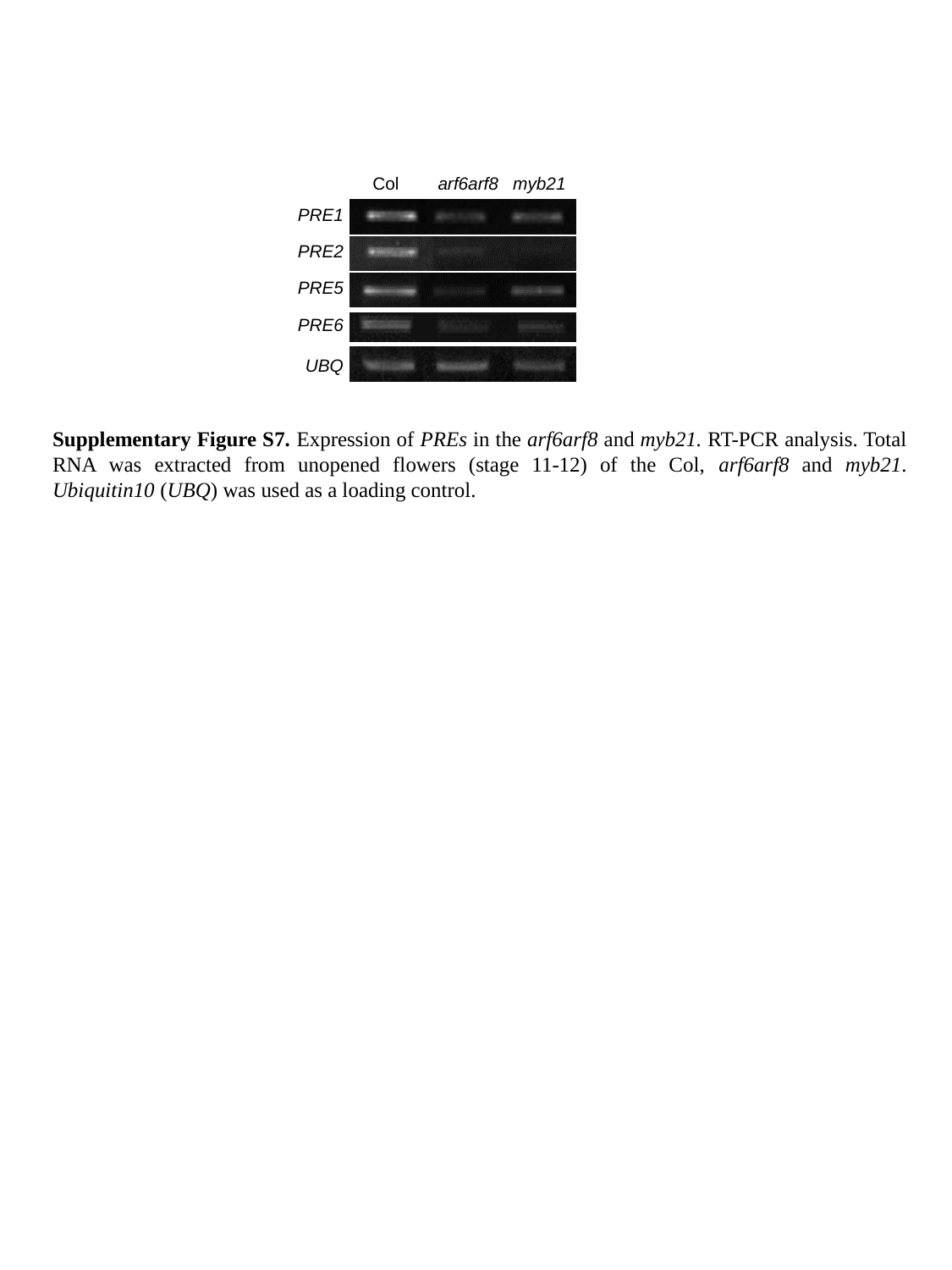

Col arf6arf8 myb21
PRE1
PRE2
PRE5
PRE6
UBQ
Supplementary Figure S7. Expression of PREs in the arf6arf8 and myb21. RT-PCR analysis. Total RNA was extracted from unopened flowers (stage 11-12) of the Col, arf6arf8 and myb21. Ubiquitin10 (UBQ) was used as a loading control.

## Slide 8
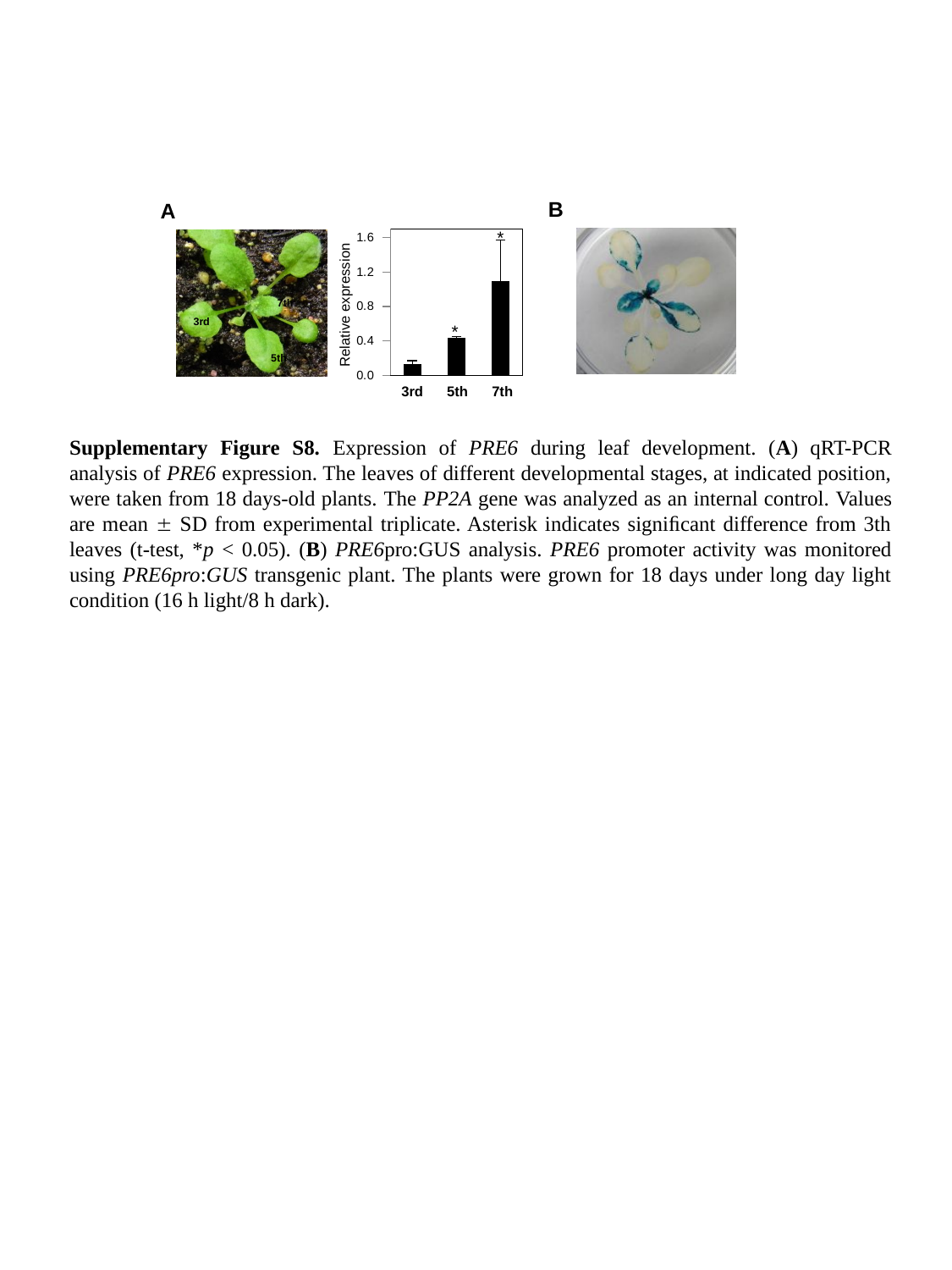

### Chart
| Category | PRE6 |
|---|---|
| 3th | 0.13260132601065117 |
| 5th | 0.43741222993019413 |
| 7th | 1.0961619529840056 |B
A
*
7th
3rd
5th
Relative expression
*
3rd
5th
7th
Supplementary Figure S8. Expression of PRE6 during leaf development. (A) qRT-PCR analysis of PRE6 expression. The leaves of different developmental stages, at indicated position, were taken from 18 days-old plants. The PP2A gene was analyzed as an internal control. Values are mean ± SD from experimental triplicate. Asterisk indicates signiﬁcant difference from 3th leaves (t-test, *p < 0.05). (B) PRE6pro:GUS analysis. PRE6 promoter activity was monitored using PRE6pro:GUS transgenic plant. The plants were grown for 18 days under long day light condition (16 h light/8 h dark).
